# Ductal Carcinoma In Situ Progression in Dog Model of Breast Cancer

**DOI:** 10.3390/cancers12020418

**Published:** 2020-02-11

**Authors:** Sulma I. Mohammed, Sagar Utturkar, Maxwell Lee, Howard H. Yang, Zhibin Cui, Nadia Atallah Lanman, GuangJun Zhang, Xavier E. Ramos Cardona, Suresh K. Mittal, Margaret A. Miller

**Affiliations:** 1 Department of Comparative Pathobiology, Purdue University, West Lafayette, IN 47907, USA; cuizhibin1985@gmail.com (Z.C.); natallah@purdue.edu (N.A.L.); gjzhang@purdue.edu (G.Z.); xramosca@purdue.edu (X.E.R.C.); mittal@purdue.edu (S.K.M.); mille188@purdue.edu (M.A.M.); 2 Center for Cancer Research, Purdue University, West Lafayette, IN 47907, USA; sutturka@purdue.edu; 3 High Dimension Data Analysis Group, Center for Cancer Research, National Cancer Institute, Bethesda, MD 20852, USA; leemax@mail.nih.gov (M.L.); yanghow@mail.nih.gov (H.H.Y.)

**Keywords:** canine, dog, mammary tumors, breast cancer, gene expression, DCIS, ADH, DCIS progression

## Abstract

The mechanisms that drive ductal carcinoma in situ (DCIS) progression to invasive cancer are not clear. Studying DCIS progression in humans is challenging and not ethical, thus necessitating the characterization of an animal model that faithfully resembles human disease. We have characterized a canine model of spontaneous mammary DCIS and invasive cancer that shares histologic, molecular, and diagnostic imaging characteristics with DCIS and invasive cancer in women. The purpose of the study was to identify markers and altered signaling pathways that lead to invasive cancer and shed light on early molecular events in breast cancer progression and development. Transcriptomic studies along the continuum of cancer progression in the mammary gland from healthy, through atypical ductal hyperplasia (ADH), DCIS, and invasive carcinoma were performed using the canine model. Gene expression profiles of preinvasive DCIS lesions closely resemble those of invasive carcinoma. However, certain genes, such as *SFRP2*, *FZD2*, *STK31*, and *LALBA*, were over-expressed in DCIS compared to invasive cancer. The over-representation of myoepithelial markers, epithelial-mesenchymal transition (EMT), canonical Wnt signaling components, and other pathways induced by Wnt family members distinguishes DCIS from invasive. The information gained may help in stratifying DCIS as well as identify actionable targets for primary and tertiary prevention or targeted therapy.

## 1. Introduction

There is evidence that all breast cancer subtypes evolve from a non-invasive ductal carcinoma in situ (DCIS) precursor stage [1]. Seldom encountered before mammographic screening became routine, DCIS now accounts for about 20% of breast cancer diagnoses [2]. Although many cases of DCIS regress or remain indolent, some may progress to invasive cancer, which creates a management dilemma, as there is no reliable molecular marker to identify those cases of DCIS destined to progress. Therefore, the current standard of care for all DCIS patients is surgery, radiation, or hormonal therapy. This management approach has good disease-free outcomes, but entails over-treatment in most cases, subjecting women to unnecessary medical and psychological side effects [3,4]. There is an urgent need to understand the natural history of DCIS and to develop methodologies to distinguish non-progressive versus progressive cases.

Elucidating the natural history of DCIS in prospective studies in women would take decades and pose ethical concerns. To address these limitations, animal models were developed with lesions that mimic DCIS histologically, including sporadic tumors in transgenic mice [5], environmentally induced cancers in immunocompromised rodents, and xenograft models [6]. However, rodent models seldom develop mammary cancer spontaneously (without experimental or genetic manipulation); therefore, they may not accurately reflect the pathogenesis of breast cancer nor reproduce many aspects of the human disease. Furthermore, mice differ considerably from humans in terms of immune system development, activation, and innate and adaptive responses. In addition, mouse mammary tumors are far less heterogeneous than human breast cancer [7]. Only 11% of the drugs that showed strong efficacy and inhibited tumor growth in mice are approved by the U.S. Food and Drug Administration (FDA) for human use [8]. Furthermore, the deleterious side effects seen in humans have not been observed in mice. Therefore, it is essential to decipher the natural history of DCIS in an animal model that develops mammary DCIS and invasive cancer spontaneously and resembles the human disease in every aspect.

Our laboratory and others have shown that there are many features common to human and canine breast cancer. As in women, and in contrast to rodent models, the mammary gland is the most common site of cancer in unspayed female dogs [9,10]. In addition, preinvasive lesions, such as atypical ductal hyperplasia (ADH) and DCIS, develop spontaneously before invasive cancer in canine mammary tissue [11,12]. Breast cancer treatments are similar in dogs and humans, and dogs and humans share many of the same breast cancer risk factors including advancing age, progesterone treatment, and obesity in early life, diet, and mutations in *BRCA* genes [13]. Canine DCIS and invasive cancer resemble human DCIS and its invasive progression with respect to histopathology, the expression of many tumor markers including estrogen receptor (ER), progesterone receptor (PR), human epidermal growth factor receptor (HER2), Ki-67, clinical outcomes, and imaging characteristics [14,15]. We have also shown that a fraction of canine DCIS were triple negative [14].

Furthermore, 50% of randomly screened asymptomatic dogs had preinvasive mammary lesions [16], and mammary DCIS often progresses to invasive cancer within one year [17]. Additionally, strong similarities exist between humans and dogs about tumor-infiltrating lymphocytes (TIL), such as the correlation between TIL numbers and mammary tumor aggressiveness, the association between the CD4+/CD8+ T cell ratio and survival rate, and the association between high Treg cell numbers and poor prognosis [18]. Given the many shared features of canine and human breast cancer and the high homology between the canine and human genome, the dog model offers an outstanding opportunity for developing breast cancer preventative and therapeutic strategies including immunoprevention. Moreover, the prevalence and rapid progression of canine DCIS provides a more efficient and cost-effective alternative to human trials for preclinical evaluation of the safety and effectiveness of cancer vaccine and other strategies.

In a canine model of breast DCIS, we investigated the transcriptome of mammary lesions along the continuum of cancer progression in the mammary gland, from healthy to ADH to DCIS to invasive cancer. The purpose of the study was to identify markers and altered signaling pathways that lead to invasive cancer and shed light on early molecular events in breast cancer progression and development. The information gained may help in stratifying DCIS as well as identify actionable targets for primary and tertiary prevention or targeted therapy.

## 2. Results

### 2.1. Gene Expression Profiles of ADH, DCIS and Invasive Cancer

**Figure 1:**
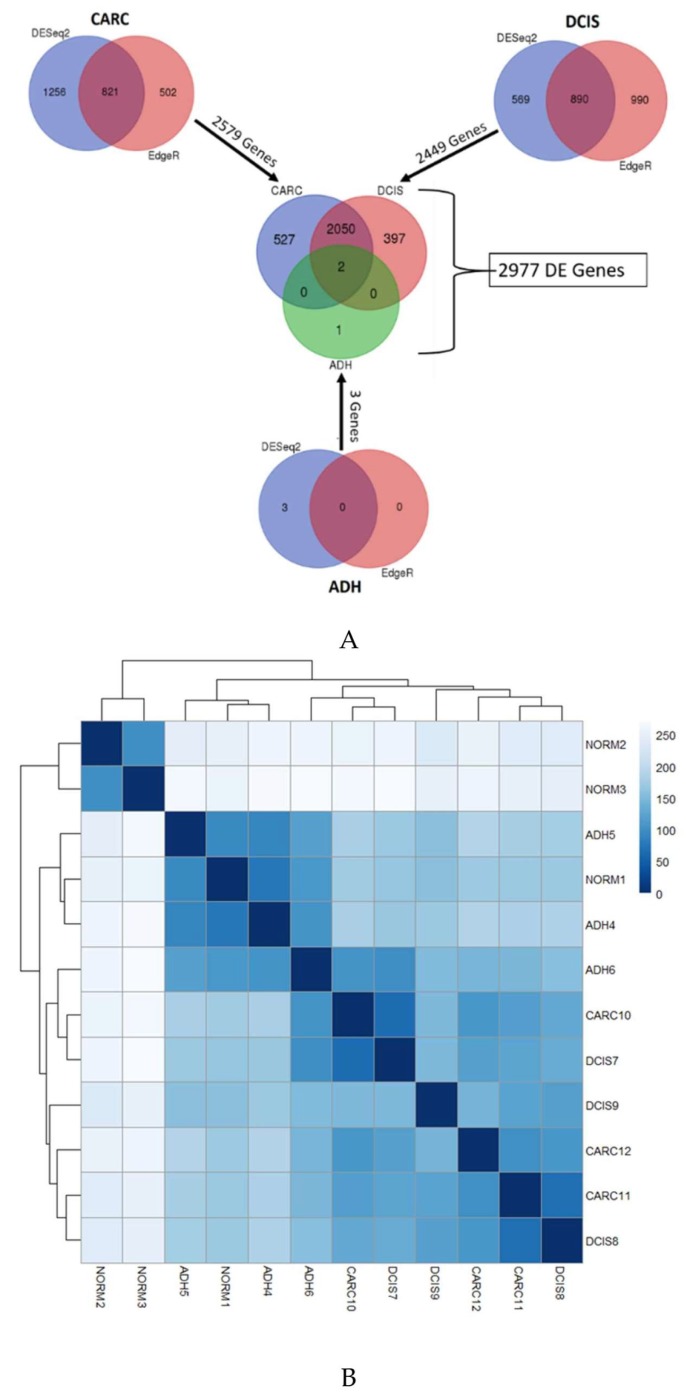
(**A**) Overlap of Differential Expression (DE) genes across three stages of cancer as determined by DESeq2 and Cufflinks methods. (**B**) Heatmap of sample-to-sample distances. A heatmap showing the Euclidian distances between samples, made with the DESeq2 transformed data after a regularized log transformation was performed. Although, not all replicates are clustered by the samples conditions, the atypical ductal hyperplasia (ADH) samples are distant from the ductal carcinoma in situ (DCIS) and invasive carcinoma (CARC).

The molecular changes associated with normal, ADH, and DCIS progressing to invasive cancer were investigated in the same mammary gland in the same dog, using next-generation sequencing. The overall mapping rate against the canine reference (CanFam3.1) was 80–96% for all samples. Table 1 shows the complete quality trimming and mapping statistics. To identify the changes associated with the progression of ADH and DCIS to invasive mammary cancer as compared to normal, we evaluated the differential gene expression results derived from the union of DESeq2 [19] and EdgeR ([20,21]) software at a threshold of False Discovery Rate (FDR) < 0.05. A total of three atypical ductal hyperplasia (ADH), 2449 ductal carcinoma in situ (DCIS), and 2579 invasive cancer (CARC) differentially expressed genes were determined. A total of 527 differentially expressed genes were exclusively present in invasive cancer, while 397 differentially expressed genes were exclusive to DCIS, and 2050 genes were expressed in both DCIS and invasive cancer (Figure 1A). The number of differentially expressed genes across samples along with results from sample distribution plots suggests that ADH is closer to normal mammary tissue while substantial transcription changes are acquired in DCIS and invasive cancer (Figure 1B).

### 2.2. Identification of Genes Associated with DCIS Progression to Invasive Cancer

**Figure 2:**
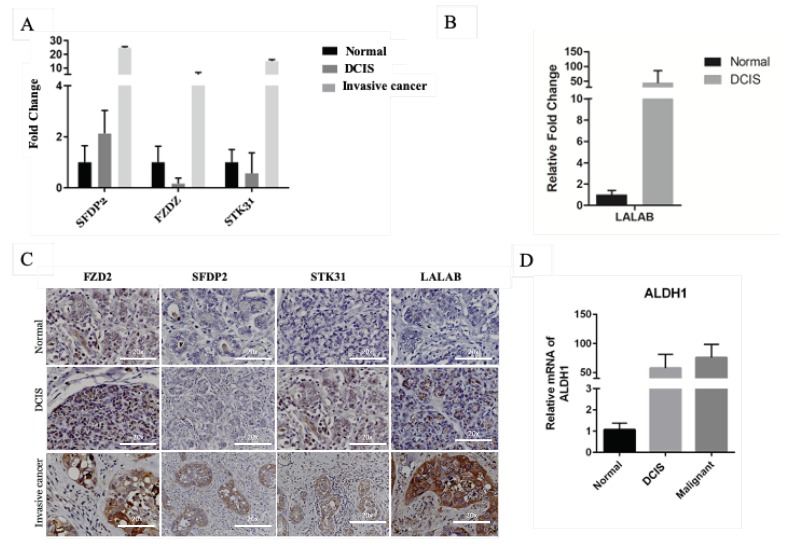
Expression of mRNA and protein of FZD2, SFDP2, STK31, and LALAB in canine normal, DCIS, and invasive cancer. (**A**) mRNA expression of FZD2, SFDP2, and STK31 in canine normal, DCIS, and invasive cancer tissues. (**B**) mRNA expression of LALAB. (**C**) Protein expression of FZD2, SFDP2, STK31, and LALAB in canine normal, DCIS, and invasive cancer. (**D**) mRNA expression of ALDH1.

To identify molecular changes associated with DCIS progression to invasive cancer, the list of 2977 differentially expressed genes was filtered to detect 364 (DCIS log2fold-change ±3) and 28 (DCIS log2fold-change ±4) protein-coding genes (Table 2). These genes included *KLRD1, S100A12, EPHA5, SFRP2, FZD2, STK31,* and *PCDH15*. We selected genes involved in the *Wnt* singling pathway, which is known to contribute to breast cancer progression Frizzled receptors (*FZD2*), secreted frizzled-related protein 2 (*SFRP2*), and genes that only expressed during certain physiological changes such spermatogenesis, serine/threonine kinase 31 (*STK31*), and lactation, alpha-lactalbumin (*LALAB*). To verify that the selected genes were over-expressed in canine mammary cancer tissues, we performed immunohistochemistry on normal, DCIS, and invasive cancer in another cohort of dogs. Among the selected genes, RNA and proteins of FZD2, SFRP2, and STK31 were significantly over-expressed in DCIS and invasive cancer (Figure 2A,C). FZD2 and its ligands *Wn*t5a/b (*Wnt* signaling pathway) were elevated in many cancers including breast cancer, and their expression correlated with epithelial–mesenchymal transition (EMT), which is a process that allows cancer cells to infiltrate surrounding tissues and metastasize to distant sites [22]. EMT is associated with cancer stem cells, which is a subpopulation of cancer cells with self-renewal and differentiation capacity, which play an essential role in tumor initiation, progression, metastasis, recurrence, and therapy resistance. Surface marker CD4 and intracellular marker Aldehyde dehydrogenase 1 family member A1 (ALDHA1) enzyme identify these cells [23]. To confirm that DCIS transition to invasive cancer associated with EMT in canine, we evaluated the expression of *ALDHA1* RNA in these lesions. We found that ALDH1A expression increased in DCIS and invasive cancer (Figure 2D). Another protein that is upregulated in canine DCIS and invasive cancer is LALBA, although it did not pass the threshold of FDR < 0.05 in our RNAseq data (Figure 2B,C).

**Figure 3:**
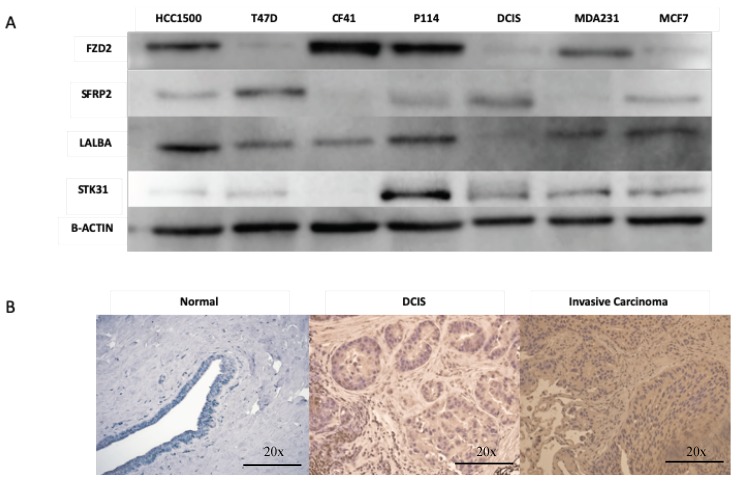
(**A**) Expression of FZD2, SFRp2, LALAB, and STK31 proteins in malignant human cell lines (HCC1500, T47D, MDA231, and MCF7), human carcinoma in situ cell line (DCIS.com), and canine cell line (CF41 and P114). (**B**) Expression of LALAB protein in women normal, DCIs, and invasive cancer tissues.

**Figure 4:**
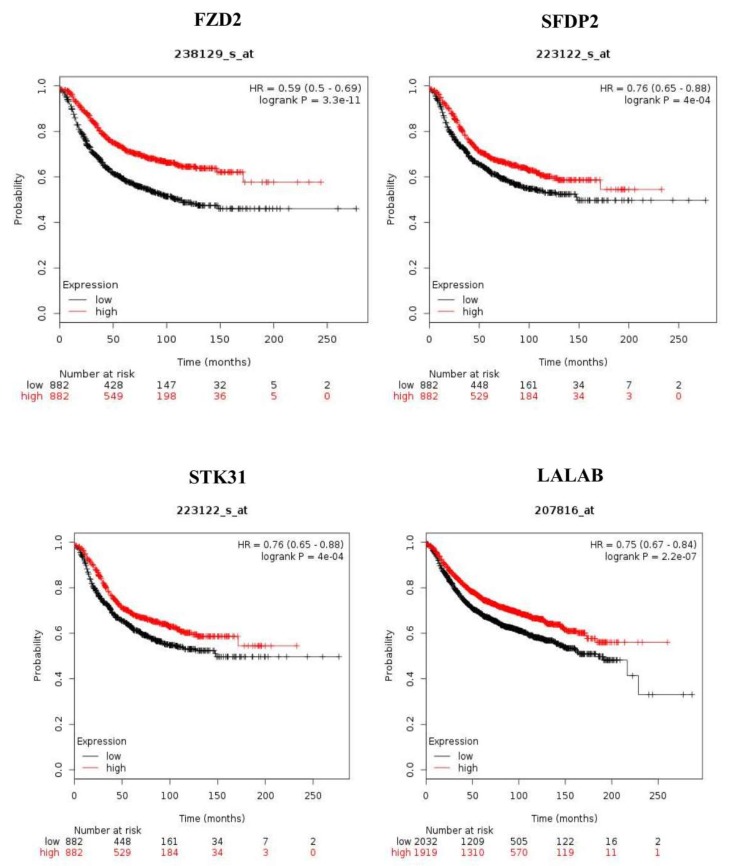
Kaplan-Meier estimates of the relapse-free survival of patients using the four genes. The Kaplan-Meier estimator plots were used to visualize the survival probabilities for the low-risk and high-risk groups, as determined using the median risk score for each dataset.

Furthermore, we tested the expression of FZD2, SFRP2, STK31, and LALBA proteins in canine mammary (CF41 and P114) and human breast cancer cells lines (HCC1500, T47D, DCIS.com, MDA231, and MCF7) (Figure 3A and Figure S1A–E). FZD2 protein is expressed in both canine cell lines (CF41 and P114) and in both triple-negative human cell lines (HCC1500 and MDA231). LALBA was over-expressed in DCIS and invasive cancer compared to normal in human tissues (Figure 3B). We evaluated the significance of *FZD2* and *STK31* by matching them against genes’ expression of human breast cancer from The Cancer Genome Atlas (TCGA) (https://cancergenome.nih.gov). Kaplan–Meier survival analysis of gene expression (*n* = 1768 patients) for *FDZ2*, *SFRP2 STK31*, and *LALBA* genes revealed that the high expression of these genes correlated significantly with shorter relapse-free survival of patients (Figure 4 A–D).

### 2.3. Genes Expression Similarities between Canine and Humans DCIS and Invasive Cancer

**Figure 5:**
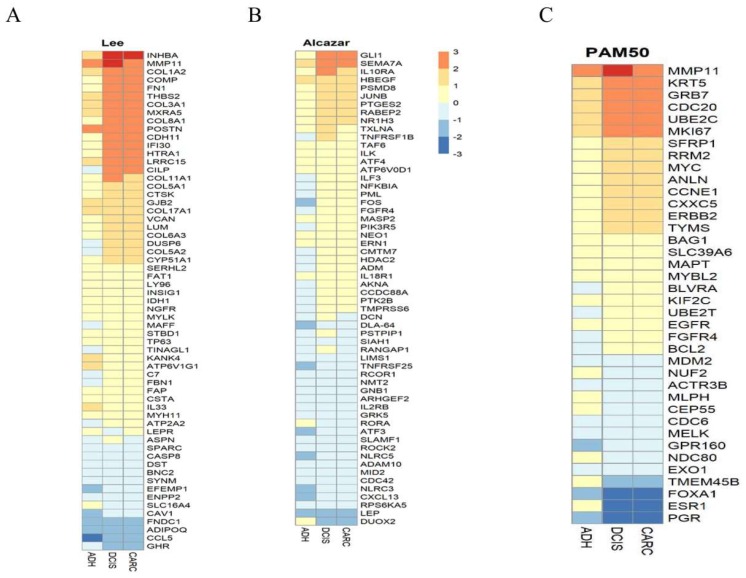
(**A**) Hierarchical clustering of 72 genes shared between canine and women ADH, DCIS, and invasive cancer. (**B**) Heatmap of essential human immune genes in canine normal, DCIS and carcinoma samples. (**C**) Heatmap of PAM50 gene set identified in canine samples.

Differentially expressed canine genes had significant overlap with similar DCIS gene signature reported by others in humans [24,25,26,27]. We examined the 74 genes identified by Lee et al., who employed microarray technology for the identification of differentially expressed genes in clinical samples (*n* = 77 divided across five groups) of human DCIS and cancer. The data showed that the 74 genes classified DCIS versus invasive cancer, and of those, 49 of the identified genes overlapped with one or more of the previous studies above. We found that all 74 genes identified by Lee et al. [27] are expressed in our study (Figure 5A); however, only 13 (FDR 0.05) or 15 (FDR 0.10) genes were significantly differentially expressed in our study. These 13 genes were also expressed at different levels in DCIS and invasive cancer by other studies [25,26]. Among the over-expressed genes were *INHBA, THBS2, MXRA5, MMP11, COL1A2, COL3A1, COMP, COL8A1, FN1, CDH11,* while *CCL5, ADIPOQ, FNDC1*, and *GHR* are under-expressed. These findings suggest a substantial similarity between canine and humans.

To investigate the immune escape during DCIS progression to invasive cancer, we examined our gene expression data against data generated by Alcazar et al. [28], who investigated potential mechanisms of immune escape in breast cancer and identified top differentially expressed and high variance immune-related genes in human DCIS. Although immune cells were not profiled in this study, we examined essential human immune genes in canine carcinoma, which are depicted as a heatmap across normal, DCIS, and carcinoma samples (Figure 5B). Similarly, immune-related genes such as *HBEGF, SEMA7A, GLI1, IL10RA, JUNB, PSMD8, PTGES2, ILF3, NF-kB,* and *TNFRSF1B* were over-expressed, while *DUOX2* and *RORA* were under-expressed in DCIS and cancer compared to ADH and normal tissues in our study as well as that of Lee et al. [27].

We next compared differentially expressed genes between DCIS and invasive cancer to the most known and widely used the prosigna breast cancer progression gene signature (PAM50) assay used for intrinsic subtyping [29]. Forty of the 50 PAM50 genes were included in our dataset, and seven were significantly differentially expressed (Figure 5C). To further investigate whether the dog model resembled human breast cancer subtypes, both the PAM50 algorithm and unsupervised hierarchical clustering were used to identify the intrinsic subtypes of our dog mammary tumors. PAM50 assigned most of the canine DCIS examined in this study to the ER-, PR-, and Her-2+ subtypes.

**Figure 6:**
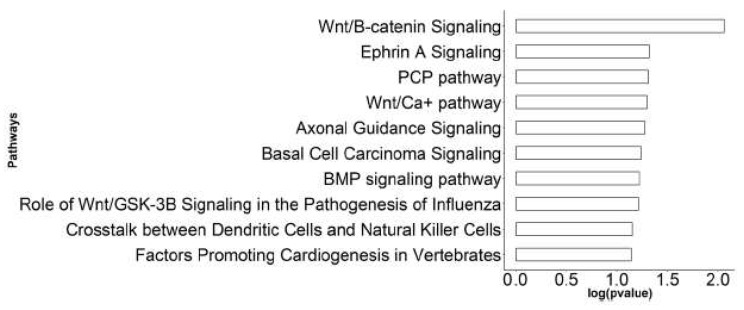
Enriched ingenuity pathway analysis (IPA) pathways of differentially expressed genes in canine tissues.

**Figure 7:**
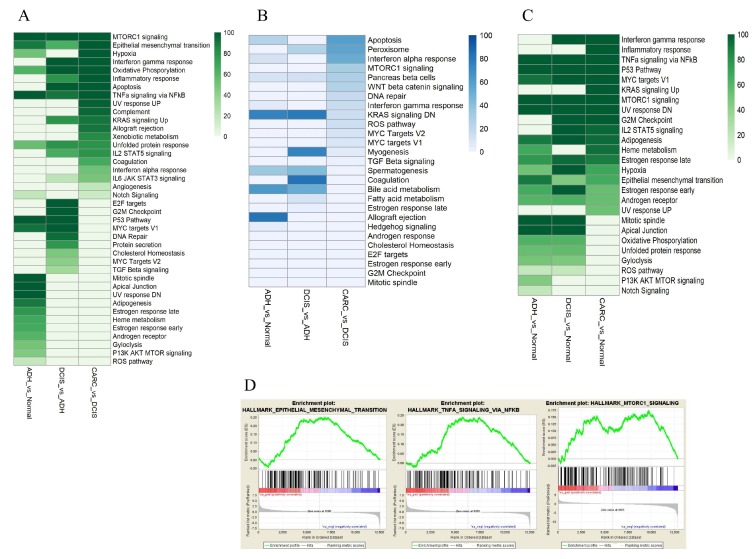
Analysis of the functional gene set enrichment in ADH, DCIS, and invasive cancer. (**A**) Gene set identified with a positive normalized enrichment score (NES) at *p*-value < 0.05 and False Discovery Rate (FDR) < 0.05. (**B**) Pathways identified with a negative NES at *p*-value < 0.05, and FDR   < 0.05. (**C**) Gene set enrichment for epithelial–mesenchymal transition, NF-KB, and MTORC1. The differential gene expression ranked by fold change. The most up-regulated genes are shown on the left while the most down-regulated genes are shown on the right. The black vertical lines indicate where the genes in the signature get set appeared. (**D**) Pathways overexpressed along the continuum in ADH, DCIS, and cancer compared to normal.

Then, we utilized ingenuity pathway analysis (IPA) (Qiagen, CA, USA) and gene set enrichment analysis (GSEA)-hallmark enrichment [30,31] of the differentially expressed genes identified at each stage. Of the IPA-enriched pathways were the *Wnt* singling pathways (canonical and non-canonical) and signaling pathways regulated by the *Wnt* pathway such as signaling pathways regulating the pluripotency of stem cells and planar cell polarity (PCP), as well as basal cell carcinoma (Figure 6). Then, we applied GSEA to test whether a member of each gene set occurs randomly toward the top or the bottom of an entire ranked list of differentially expressed genes. We employed a normalized enrichment score (NES), which reflects the degree of over-representation of a gene set at the top or bottom and takes into account the size of the gene set. In addition, we used a nominal p-value that estimates the statistical significance of NES using a gene-set based permutation procedure and FDR, which controls the proportion of false positives to identify up/down-regulated gene [30]. Among the differentially expressed genes along the progression continuum from normal to ADH to DCIS and finally to invasive cancer, more than 40 gene sets were identified with a positive NES at *p*-value <  0.05 and FDR  < 0.05 (Figure 7A), and 25 pathways were identified with a negative NES at *p*-value <  0.05 and FDR   < 0.05 (Figure 7B). Among the significantly enriched gene sets were an epithelial–mesenchymal transition, *NF-KB*, and *MTORC*1 (Figure 7C). Furthermore, pathways that include *p53*, *TNFα* via *NF-κβMTORC1*, and *MYC* targets were over-expressed along the continuum in ADH, DCIS, and cancer compared to normal (Figure 7D).

## 3. Discussion

The natural history of DCIS progression remains mostly unknown because of the surgical removal of DCIS lesions upon diagnosis. The currently accepted view of breast cancer pathogenesis assumes a linear progression in which ADH transitions through DCIS to invasive cancer and metastasis. Although epidemiologic, pathologic, and molecular data in humans and animals support the concept of evolution of invasive cancer from DCIS, in the few retrospective studies of DCIS (initially misdiagnosed as ADH) without curative treatment, only 25–50% of DCIS progressed to invasive cancer within 15–25 years [32]. Therefore, the current standard of care leads to the over-treatment of many patients. However, the factors that drive some DCIS to become invasive are not yet understood, so there is an urgent need for diagnostic markers to stratify DCIS lesions and effective targeted therapy to prevent DCIS transition to invasive cancer.

The molecular analysis and identification of markers to stratify DCIS into progressive and indolent cases ideally requires the study of DCIS as it progresses with comparison before and after the onset of invasiveness. This approach was hindered by the limited availability of human tissues from evolving lesions in the same breast, the time needed for progression (more than a decade), ethical concerns, and the lack of an animal model that faithfully represents human disease. Previously, we described the canine mammary gland and cancer as an excellent model for human disease. We have shown that ADH and DCIS in dog mammary glands resemble those of humans in every aspect, including histopathology, clinical outcomes, and imaging characteristics [14,15]. In addition, we have shown that DCIS is prevalent in asymptomatic dogs as in humans [16]. In this study, we performed transcriptomic studies of mammary lesions along the continuum of cancer progression in the same gland (normal to ADH to DCIS to invasive cancer) in the same dog. We described the molecular similarities of DCIS and invasive cancer between humans and dogs, and identified potentially unique targets and perturbed pathways that relate to DCIS progression to invasive cancer in the dog model.

The differentially expressed genes across samples along with results from sample distribution plots suggest that ADH is similar to healthy mammary tissue, but that substantial transcription changes are acquired in DCIS and invasive cancer, which is in agreement with reports that DCIS is molecularly, histopathologically, clinically, and biologically distinct from ADH [33]. In spite of this distinction, common up-regulated pathways were identified between DCIS and ADH, such as *p53* and Myc, which were shared between ADH and DCIS, but not invasive cancer, while epithelial–mesenchymal transition (EMT), *TNFα/NF-kβ*, and *MTORC1* signaling pathways were enriched throughout the cancer progression continuum from ADH to DCIS to invasive cancer. These signaling pathways are involved in cancer progression, invasion, and metastasis [34,35,36] and associated with stem cell-like features [37].

The close similarities in gene over- and under-expression between DCIS and invasive cancer have been reported with many studies unable to consistently differentiate DCIS from paired invasive cancer in humans [25,26,38]. No mutations are unique to invasive cancer in comparison to DCIS. Similarly, the gene expression profile showed a highly similar pattern in DCIS and invasive cancer. Therefore, it was suggested that the genetic program necessary for invasive progression might already exist in the preinvasive stages of breast cancer and that a final series of subtle events in DCIS drives the transition to invasive cancer [25,39]. However, Ma et al.’s study using samples from women found significant global alterations in gene expression at the ADH stage that are maintained in the later stages of DCIS and invasive cancer [25]. In another study by Porter et al., no genes were specific for invasive cancer or DCIS, and the most dramatic and consistent phenotypic changes occurred at the normal-to-in situ carcinoma transition in women [40]. However, other studies that compared the transcriptome of DCIS and invasive cancer using more robust techniques have identified stage-specific markers and a gene expression classifier that differentiate DCIS and invasive cancer [26,41]. This discordance may be because none of the described studies compared DCIS samples before and after invasiveness or with DCIS that did not give rise to invasive disease. Furthermore, when we compared our data to published data by Lee et al. [27] and Knudsen et al. [24], only 13 genes overlapped with their data, which may be due to the reasons mentioned above, the sample size differences between the two studies, and the fact that the comparison of gene expression data obtained from different array platforms results in a marginal overlap [42] and might not suggest species differences.

Recently, growing evidence suggests that the breast microenvironment, including myoepithelial cells, stroma, and extracellular matrix (ECM), plays a crucial role in the transition from DCIS to invasive cancer [43,44]. Tumor-associated myoepithelial cells have a tumor-protective effect [43]. The myoepithelial layer and basement membrane that surrounds DCIS is thought to be lost during progression, allowing tumor cells to invade the stroma and surrounding adipose tissue. We found myoepithelial cells markers, including *MMP11*, *COL1A2*, *COL3A1, COL8A1, S100A2*, and *FN1*, were over-expressed in DCIS compared to invasive cancer (Table 2) [45,46]. The expression of S100A2 is induced by transforming growth factor-β (*TGF-β*) in DICS (Figure 7A), which is a potent inducer of EMT (Figure 7C) and cancer progression. These findings support a model wherein alterations in myoepithelial cells promote the progression of DCIS to invasive cancer via TGFβ signaling activation. Activation of the *TGFβ* signaling pathway promoted the EMT, basal-like phenotypes, stemness, and invasiveness of DCIS cells.

Despite the close similarities in gene expression between DCIS and invasive cancer, we identified genes that differed significantly in expression between DCIS and invasive cancer. In our study, the differentially expressed genes in *DCIS* compared to invasive cancer are *FZD2* and *SFRP2*, which are the core WNT pathway components, *STK31*, and *LALBA*. *FZD2* is elevated in many cancers, including breast cancer, and its expression correlated with markers of EMT and cell migration through a non-canonical pathway that predicts metastasis and overall survival in patients [22]. Similarly, *SFRP2* is shown to be associated with poor prognosis concomitant with the expression of genes associated with EMT [47]. In agreement with the above findings, the pathway analysis of the gene expression data in this study showed enrichment of WNT (both canonical and non-canonical) pathways, the planar cell polarity (PCP) pathway, and signaling pathways regulating the pluripotency of stem cells in DCIS (generally induced by WNT signaling). The Wnt signaling pathway is known to promote cancer progression, metastasis, and is involved in EMT [48]. EMT is essential in tumor invasion because it reduces cell–cell adhesion and increases cell motility. The non-canonical PCP pathway as mentioned above exerts an essential role in cell differentiation by regulating critical components of the cytoskeleton that lead to cell shape and motility changes [49]. These processes are primary events in the multi-step progression from DCIS to invasive cancer.

On the other hand, cancer *STK31*, a cancer-testis antigen (CTA), plays crucial roles in human cancer through regulation of the cell cycle, and its over-expression increases cell migration and invasiveness, whereas its depletion induces apoptosis [50]. This molecule is expressed in the human germline, but it is not expressed in most adult tissues, where it is restricted to the testis and various malignancies. CTA expression is highly increased in DCIS of the breast, particularly those that are ER-negative [51]. As a result of their restricted expression pattern, these proteins are immunogenic in cancer patients and are considered essential targets of cancer vaccines.

LALBA expression was high in DCIS and invasive cancer, but it did not pass our FDR threshold of <0.05. Our interest in α-lactalbumin stems from the fact that it is expressed in mammary epithelial cells only during lactation [52] but expressed at high levels in the majority of human breast carcinomas [53]. α-Lactalbumin as a vaccine target mediated protection against the development of murine breast cancer in the absence of any detectable autoimmune-induced breast inflammation.

Human breast cancer consists of four subtypes: luminal A and B, triple-negative, and Her-2 positive. Previously, we reported that one-third of canine DCIS and tumors are immunohistochemically negative for hormonal receptors. In this study, PAM50 assigned most of the tumors examined in this study to the HER-2 breast cancer subtype, which is the subtype most commonly associated with ER-negative and HER-2 positive subtype, corroborating the similarities between our dog model and human breast cancer.

## 4. Materials and Methods

### 4.1. Clinical Specimens

The Purdue animal care and use committee approved all animal experiments. For this pilot study, healthy, ADH, DCIS, and invasive cancer samples were collected from the same progressing lesions in mammary glands from the same dogs (*n* = 3). Tissue blocks were reviewed by a board-certified veterinary pathologist to confirm the diagnosis and define lesions for dissection. Immunohistochemistry with antibodies to estrogen receptor (ER)-α, progesterone receptor (PR), and human epidermal growth factor-2 (HER-2) was used to determine hormonal expression status.

### 4.2. RNA Isolation

Five serial sections of 5-µm thickness were stained; then, normal or abnormal cells were microdissected from each lesion. Total RNA was extracted from each lesion in 300 μL of lysis buffer with 50 μg of proteinase K using MasterPure Complete DNA and RNA Purification Kit (Epicentre Biotechnologies, Madison, WI, USA) according to manufacturer instructions. Genomic DNA contamination was removed by treatment with DNase I (10 U/mL; Ambion, Inc, Austin TX, USA 78744). Then, the RNA was dissolved in RNase-free water and quantified using NanoDrop 1000 spectrophotometer (Thermo Fisher Scientific, Waltham, MA, USA) at 260 nm.

### 4.3. Next-Generation Sequencing

Individual RNA samples were submitted to Otogenetics (Norcross, GA, USA) for RNA-Seq analysis. Briefly, the integrity and purity of the RNA samples were assessed using the Agilent Bioanalyzer and OD260/280 (Agilent, Santa Clara, CA, USA). rRNA was depleted from each sample using the Ribo-Zero rRNA Removal Kit (Epicentre, Madison, WI) according to the manufacturer’s instructions. cDNA was generated using the Smart PCR cDNA kit (Clontech Laboratories, Mountain View, CA, USA) from 100 ng of total RNA, and adaptors were removed by digestion with Rsa I. This method used a low cycle number PCR to preferentially amplify poly (A+) RNA via a modified oligo (dT) primer. The resultant cDNA was fragmented via sonication (Covaris, Inc., Woburn, MA, USA), profiled via Agilent Bioanalyzer, and subjected to Illumina library preparation using NEBNext reagents (New England Biolabs, Ipswich, MA, USA). The quality, quantity, and size distribution of the Illumina libraries were determined using an Agilent Bioanalyzer 2100 (Agilent, Santa Clara, CA, USA). Then, the libraries were submitted for Illumina HiSeq2000 sequencing as per the manufacturer’s recommendations. Paired-end 90 or 100 nucleotide reads were generated, checked for data quality using FASTQC (Babraham Institute, Cambridge, UK), and subjected to data analysis using the DNAnexus platform (DNAnexus, Inc, Mountain View, CA, USA). Transcript-level quantitation was per the DNAnexus White Paper (version 1.1, April 19, 2010, DNAnexus, Mountain View, CA, USA) on RNA-Seq/3SEQ Transcriptome.

### 4.4. Bioinformatics Analysis

Sequence data quality was determined using FastQC (version 0.11.2, Babraham Institute, Cambridge, UK). Quality trimming and filtering were performed using TrimGalore (version 0.4.4). Illumina adapter sequences, any bases with Phred score less than 30, and reads shorter than 50 base pairs were removed during quality control. Quality trimmed reads were mapped against the Canis familiaris reference genome (CanFam3.1) using STAR aligner [54]. Read counts for each gene feature each replicate were determined using the HTSeq tool [55] and the combined count matrix for all replicate was generated using a custom Perl script. For Differential Expression (DE) analysis, genes with zero counts across all replicates were discarded. DE analysis of cancer and healthy samples (ADH versus Normal, DCIS versus Normal, and invasive carcinoma (CARC) versus Normal) was carried out using ‘R-Bioconductor’ package DESeq2 ([19]) and EdgeR ([20]). Since the sequencing of replicates was performed in batches, a batch correction was applied by providing a custom experiment design matrix in DESeq2 and EdgeR analysis. Genes with a False Discovery Rate (FDR) less than 0.05 (i.e., 95% confidence interval) were denoted as DE genes. The Venn diagram denotes a subset of genes that are common across two methods (DESeq2 and EdgeR) and cancer stages (ADH, DCIS, and CARC). A list of genes that are modulated in DCIS and CARC conditions in human models were obtained from the literature survey, and their expression profile in current data is visualized as Heatmaps. Heatmaps are generated by plotting the log2FoldChnage using R package heatmap.

### 4.5. Pathway Enrichment Analysis

Pathway analysis was performed using the Ingenuity pathway Analysis (IPA) and gene set enrichment analysis (GSEA). Enriched pathways and gene sets (*p*-value < 0.05) associated with cancer stages (ADH, DCIS, and CARC) were determined. 

### 4.6. Quantitative Real-Time RT-PCR (qRT-PCR)

Total RNA was isolated from fresh-frozen healthy, benign (ADH, DCIS), and invasive cancer using Trizol reagent (Invitrogen Life Technologies, Carlsbad, CA, USA) according to manufacturer instructions. The RNA concentration was determined using the NanoDrop 1000 Spectrophotometer (Thermo Fisher Scientific, Waltham, MA, USA). Reverse transcription was performed using Transcriptor First Strand cDNA synthesis kit (Roche Diagnostics, Indianapolis, IN, USA) from 1 μg of total RNA and the combination of anchored-oligo (dT) and random hexamer primers according to the manufacturer’s instructions. Gene expression analysis was performed with qRT-PCR using the LightCycler 480 SYBR Green I Master (Roche Diagnostics, Indianapolis, IN, USA) and primers. All reactions were performed in triplicate, and the relative expression of target mRNA in each sample was normalized with that of mean *GAPDH* abundance. The primers were purchased from Integrated DNA Technologies, Inc. (Coralville, IA, USA).

### 4.7. Immunohistochemistry

Paraffin sections, 5-µm thick, were mounted on positively charged Super-frost slides (Fisher Scientific, Chicago, IL, USA), which were then deparaffinized and rehydrated in graded alcohols and subjected to immunohistochemistry according to the manufacturer’s protocol (Biocare Medical, Concord, CA, USA). In brief, the hydrated sections were heated in a Biocare Gene retrieval steamer 45 min in 10 mM of citrate buffer, pH 6.0, to unmask the epitopes. Then, 3% hydrogen peroxide was used to block the endogenous peroxidase activity. Nonspecific binding was blocked with background polisher, and slides were incubated for 10 min at room temperature. Then, the slides were incubated for 30 min with primary mouse monoclonal antibody or rabbit polyclonal antibodies as described. Then, the primary antibody complexes were visualized with a biotin-labeled secondary antibody (Universal Goat Link) for 15 min; and finally, with horseradish peroxidase (HRP) for 15 min, reacted for 5 min with 3, 3’-diaminobenzidine tetrahydrochloride, and counterstained with hematoxylin, dehydrated, and mounted. We treated negative control slides with no antibody.

### 4.8. Western Blot Analysis

Proteins were prepared by using Radioimmunoprecipitation assay buffer (RIPA) (0.5 m Tris/HCl, pH 7.4, 1.5 m NaCl, 2.5% deoxycholic acid, 10% NP-40, 10 mm EDTA). Protein concentrations were determined using a Pierce BCA Protein Assay Kit according to the manufacturer’s instructions (Thermo Fisher Scientific). Ten micrograms of each sample protein was subjected to SDS/PAGE and transferred to nitrocellulose paper. The blots were reacted sequentially with primary antibodies: HRP-conjugated goat anti-rabbit immunoglobulin (IgG) or goat anti-mouse IgG, and visualized with diaminobenzidine.

### 4.9. Kaplan Meier Srvivial Analysis

To determine the significance of *FDZ2*, *SFRP2*, *STK31*, and *LALBA* genes that we identified in canine DCIS progression in women with breast cancer survival, we performed Kaplan–Meier survival analysis using TCGA data and the publically available Kaplan–Meier plotter. The cut-off for the analysis was set at the auto select best cut-off. It is 172, 8383, and 9 for FZD2, SFRP2, and STK31, respectively.

### 4.10. Statistical Analysis

All data are presented as means ± standard deviation (SD). Statistical calculations were performed with Microsoft Excel analysis tools. Differences between individual groups were analyzed by paired *t* test. *p*-values of < 0.05 were considered statistically significant.

## 5. Conclusions

We report gene expression profiles along the cancer progression continuum from ADH to DCIS to invasive cancer in the canine model of breast cancer. In previous studies, we showed that canine DCIS and invasive cancer resemble human DCIS and its invasive progression to histopathology, the expression of many tumor markers, and clinical outcomes, as well as imaging characteristics. The results of this study suggest that alteration of the *Wnt* signaling pathway may play a role in the progression of DCIS to invasive cancer, similar to data reported in humans. In addition, we have identified gene markers, such as STK31 and α-lactalbumin, that may serve as vaccine targets for the prevention of DCIS progression. With its morphological, diagnostic imaging, and transcriptomic similarities to mammary DCIS and invasive carcinoma in women, the canine model could have a transformative impact on therapeutic and preventative strategies for breast cancer.

## Figures and Tables

**Table 1:** Quality trimming and mapping statistics. A number of total reads, quality filtered reads, and overall mapping rate for each replicate and each sample are described.

Sample Group	Replicate Name	Filename	Total Reads	Quality Control Reads	% Reads Passing Quality Control	Reads went into mapping	Total Reads Mapped	Overall Mapping Rate
NORM	NORM1	NORM1T_1.fastq	15,190,966	12,695,551	83.57	25,389,212	21,810,532	85.90
		NORM1T_2.fastq	15,190,966	12,695,970	83.58			
	NORM2	NORM2T_1.fastq	48,392,239	48,392,019	100.00	96,783,168	93,500,856	96.61
		NORM2T_2.fastq	48,392,239	48,391,804	100.00			
	NORM3	NORM3T_1.fastq	55,636,922	55,636,682	100.00	111,272,404	106,104,220	95.36
		NORM3T_2.fastq	55,636,922	55,636,437	100.00			
ADH	ADH4	ADH4T_1.fastq	12,072,501	9,985,381	82.71	19,969,542	16,901,852	84.64
		ADH4T_2.fastq	12,072,501	9,985,596	82.71			
	ADH5	ADH5T_1.fastq	19,656,483	16,956,030	86.26	33,910,162	29,749,778	87.73
		ADH5T_2.fastq	19,656,483	16,956,364	86.26			
	ADH6	ADH6T_1.fastq	10,597,768	9,232,178	87.11	18,461,792	15,062,516	81.59
		ADH6T_2.fastq	10,597,768	9,232,503	87.12			
DCIS	DCIS7	DCIS7T_1.fastq	15,265,327	12,725,074	83.36	25,447,108	20,483,162	80.49
		DCIS7T_2.fastq	15,265,327	12,725,528	83.36			
	DCIS8	DCIS8T_1.fastq	21,202,092	17,943,923	84.63	35,886,362	32,592,522	90.82
		DCIS8T_2.fastq	21,202,092	17,944,297	84.63			
	DCIS9	DCIS9T_1.fastq	17,680,467	15,266,076	86.34	30,530,820	27,452,402	89.92
		DCIS9T_2.fastq	17,680,467	15,266,383	86.35			
CARC	CARC10	CARC10T_1.fastq	11,964,706	9,594,034	80.19	19,186,476	16,066,992	83.74
		CARC10T_2.fastq	11,964,706	9,594,135	80.19			
	CARC11	CARC11T_1.fastq	12,749,664	10,291,360	80.72	20,581,584	18,862,732	91.65
		CARC11T_2.fastq	12,749,664	10,291,519	80.72			
	CARC12	CARC12T_1.fastq	13,512,440	10,072,537	74.54	20,143,324	18,112,260	89.92
		CARC12T_2.fastq	13,512,440	10,072,720	74.54			

**Table 2:** List of 28 DE protein-coding genes with log2FC ±4 in DCIS-CARC transition. “De in stage” column describes the cancer stage(s) in which the gene in DE and log2FC value denotes the average log2FC value by methods DESeq2 and EdgeR. Most of the genes listed with “NA” are uncharacterized proteins.

Gene ID	Gene Name	DE in Stage	DCIS log2FC	CARC log2FC	Gene Description
ENSCAFG00000000473	*TRHDE*	CARC DCIS	−4.20	−3.93	thyrotropin releasing hormone degrading enzyme
ENSCAFG00000002606	NA	ADH CARC DCIS	−4.88	−4.83	NA
ENSCAFG00000002714	*EPHA5*	CARC DCIS	−4.00	−3.92	EPH receptor A5
ENSCAFG00000002795	*STK31*	CARC DCIS	−4.14	−3.93	serine/threonine kinase 31
ENSCAFG00000006357	*CNTN3*	CARC DCIS	−4.82	−4.72	contactin 3
ENSCAFG00000008210	*GREM1*	CARC DCIS	4.75	4.73	gremlin 1, DAN family BMP antagonist
ENSCAFG00000008337	*TMPRSS15*	CARC DCIS	−4.06	−3.98	transmembrane protease, serine 15
ENSCAFG00000008353	*SFRP2*	CARC DCIS	5.20	5.18	secreted frizzled related protein 2
ENSCAFG00000009842	*OPCML*	CARC DCIS	−4.28	−3.76	opioid binding protein/cell adhesion molecule like
ENSCAFG00000010878	*TCERG1L*	CARC DCIS	−4.32	−3.93	transcription elongation regulator 1 like
ENSCAFG00000013453	*NA*	CARC DCIS	−4.23	−3.83	NA
ENSCAFG00000013459	*KLRD1*	CARC DCIS	−4.44	−4.39	Natural killer cells antigen CD94
ENSCAFG00000013703	*PTH2R*	CARC DCIS	−4.06	−3.91	parathyroid hormone 2 receptor
ENSCAFG00000013753	*NA*	CARC DCIS	−4.48	−4.26	NA
ENSCAFG00000014134	*FZD2*	CARC DCIS	4.41	4.35	frizzled class receptor 2
ENSCAFG00000015538	*PCDH15*	CARC DCIS	−4.30	−4.07	protocadherin related 15
ENSCAFG00000017227	*GABRG2*	CARC DCIS	−4.03	−3.79	gamma-aminobutyric acid type A receptor gamma2 subunit
ENSCAFG00000019105	*NA*	CARC DCIS	−4.00	−3.66	NA
ENSCAFG00000023324	*S100A12*	CARC DCIS	4.15	4.11	S100 calcium binding protein A12
ENSCAFG00000023449	*KRT13*	CARC DCIS	4.62	4.62	keratin 13
ENSCAFG00000025443	*NA*	CARC DCIS	−4.13	−4.14	NA
ENSCAFG00000028937	*NA*	CARC DCIS	−4.21	−3.90	NA
ENSCAFG00000029431	*SYNPR*	CARC DCIS	−4.51	−4.09	Synaptoporin
ENSCAFG00000029470	NA	CARC DCIS	4.28	4.24	NA
ENSCAFG00000030453	*FAM19A1*	CARC DCIS	−4.14	−3.86	family with sequence similarity 19 member A1, C-C motif chemokine like
ENSCAFG00000031931	*HIST1H2BJ*	CARC DCIS	4.16	4.10	histone cluster 1 H2B family member j
ENSCAFG00000032610	NA	CARC DCIS	−4.04	−3.91	NA
ENSCAFG00000032615	NA	CARC	4.36	4.11	NA

## Data Availability

All datasets used and analyzed during the current study are available from the corresponding author on reasonable request.

## Supplementary Materials

The following are available online at https://www.mdpi.com/2072-6694/12/2/418/s1, Figure S1: Expression of FZD2 (**A**), SFRp2 (**B**), LALAB (**C**), STK31(**D**) and B-actin(**E**) proteins in malignant human cell lines (HCC1500, T47D, MDA231 and MCF7), human carcinoma in situ cell line (DCIS.com), and canine cell line (CF41 and P114).
